# Determinants of Satisfaction with Services, and Trust in the Information Received in Community Pharmacies: A Comparative Analysis to Foster Pharmaceutical Care Adoption

**DOI:** 10.3390/healthcare9050562

**Published:** 2021-05-11

**Authors:** Elena Druică, Rodica Ianole-Călin, Cristian Băicuș, Raluca Dinescu

**Affiliations:** 1 Faculty of Business and Administration, University of Bucharest, 030018 Bucharest, Romania; rodica.ianole@faa.unibuc.ro; 2 Department of Internal Medicine, Carol Davila University of Medicine and Pharmacy, 050474 Bucharest, Romania; cristian.baicus@umfcd.ro; 3 Department of Quality Assurance, University of Bucharest, 030018 Bucharest, Romania; raluca.dinescu@unibuc.ro

**Keywords:** patient satisfaction, community pharmacy services, trust, information asymmetry, patients’ perception, pharmaceutical care

## Abstract

Patient’s satisfaction with community pharmacy services, and patients’ trust in the information received in community pharmacies are important drivers of pharmaceutical care adoption. An online questionnaire assessing patient satisfaction with the services received in pharmacies and trust in the pharmacist’s advice, along with their determinants, was administered to 343 Romanian chronic and non-chronic patients. Using various statistical tests, exploratory factor analysis, and robust regression we explored determinants of satisfaction and trust. We found that satisfaction with services is predicted by pharmacists’ attitude (β = 631, *p* < 0.001), low waiting time (β = 0.180, *p* < 0.001), affordable cost of the drugs (β = 0.09, *p* = 0.009), and drug availability (β = 0.157, *p* < 0.001). At the same time, trust in the information received is driven by pharmacists’ attention (β = 0.610, *p* < 0.001), whether the patient received precautionary information (β = 0.425, *p* < 0.001), low waiting time (β = 0.287, *p* < 0.001), and whether the respondent is a chronic patient or not (non-chronic patients express more trust, β = 0.328, *p* = 0.04). Our study expands the existing paradigm that sees trust as a simple predictor of satisfaction by showing that trust and satisfaction are predicted by different variables, and thus they should be addressed using different strategies. In fact, we found that they share only one predictor—waiting time, highly significant in both cases. Our findings show that, without prioritizing trust in the information received in community pharmacies to reduce information asymmetry between patient and pharmacist, the focus only on patient satisfaction may lead to a scenario in which community pharmacies will end up to be better integrated in the business sector and not in the public health system.

## 1. Introduction

Community pharmacies, broadly defined as pharmacies located outside hospitals, have a dual nature, being both business-oriented and involved in providing public health care [1]. Recently, regulatory initiatives worldwide have emphasized the latter dimension and recommended a better integration of community pharmacies in health care distribution. This is a timely approach considering the increased demand for primary care [2], demand that could be addressed to a higher degree by using the expertise of pharmacists, a category that ranks, as number of healthcare professionals, immediately after physicians and nurses [3]. Despite the benefits, there seems to be slow progress for community pharmacies in Europe in transitioning to pharmaceutical care (as measured through a behavioral pharmaceutical care score for 2006 and 2013, [4]), with less available evidence for Central and Eastern European countries, including Romania.

Beyond the first step of developing a legal framework that allows community pharmacies to provide advanced health services and interventions focused on patient care, other variables may influence the implementation of pharmaceutical care: lack of time and material resources [5], lack of coordination with other health professionals [6], and pharmacists’ and patients’ resistance to change [7,8]. Our paper examines patient’s views that may impact the development of pharmaceutical care in Romania and it specifically explores determinants of patient’s satisfaction with community pharmacy services and determinants of patient’s trust in the information received in pharmacies.

Patient satisfaction is a multidimensional concept, still lacking complete construct consistency [9], inspired by a diverse set of marketing and healthcare theories. We take as a reference the seminal definition of Linder-Pelz that views patient satisfaction as the ”positive evaluations of distinct dimensions of the health care” [10]. In this vein, patient satisfaction with pharmacy services informs quality management procedures at all levels of the health care system [11], increases patient adherence to medication (e.g., diabetes, [12]), and improves patient education regarding their own condition (e.g., the importance of home blood pressure monitoring, [13]) or public health issues in general [14].

Nonetheless, there is also a debate around the drawbacks of using satisfaction as a primary outcome measure [15]. One such negative consequence is the view of satisfaction as “a popularity contest” [16] and not as a quality indicator, feeding the misperception that a pleasant interaction with patients is enough to generate satisfaction. However, developing pharmaceutical care requires more than “being nice to the patient” [5]. Without actively acknowledging this fact there is a major risk to remain in a product-oriented paradigm in which patients can be satisfied with pharmacy services, without further trusting the information received. Such a status-quo bias next to the pharmacy services’ commercial dimension may act as a barrier in enforcing pharmacy counseling practices and patient-centered care [17], especially if the attention stays only on patient satisfaction and ignores the cultivation of trust. Given the uncertainty entailed by pharmacological interventions on patients [18] and the risks related to adverse effects of drugs [19], patient safety developed as a major concern over the years [20]. This makes patient trust in the information received from pharmacists one of the most significant drivers of pharmaceutical care adoption [21]. Here, we consider patient trust as the “reassuring feeling of confidence or reliance in the physician and the physician’s intent” [22], with reference to the pharmacist. The variable is a combination of interpersonal trust (towards the health professional category) and social trust (towards the health system and institutions) [23]. 

Previous research on community pharmacies favors service satisfaction as a major theme of interest, in descriptive [24,25,26] and correlational studies. Service promptness, pharmacist attitude, medication counseling, pharmacy location, waiting area [27], and attention received [28] usually determine patient satisfaction. Socio-demographics (e.g., gender, marital status, health status, age, educational level, ethnicity, income, employment, and health insurance, [27,29]) have contextual influence, and they do not predict satisfaction in all settings [30]. Overall, satisfaction relates more to business aspects, such as environment quality and staff’s attitude [31], and less with aspects related to the pharmaceutical service content. This suggests why trust is not a central issue in this literature niche, and it is treated just like one of the many potential determinants of satisfaction.

The pharmaceutical care approach reframes pharmacy services from transactions to consultations. Consequently, trust in the advice received becomes paramount as it involves an implicit preoccupation for minimizing information asymmetry in the patient–pharmacist relationships [23]. Information asymmetry, defined as the situation where one holds more relevant information than another, is a major concern in healthcare [32]. While it is a must for the pharmacist to have more information than the patient (expressed as professional expertise), a genuine focus on patient counseling would gradually induce a decrease of this informational gap (expressed as patient empowerment). Conversely, an increase in information asymmetry may consolidate the monopoly of knowledge, leading to a power imbalance when services are provided.

Our paper explores the drivers of satisfaction and trust. Even if previously the concepts have been examined in interaction, the results do not follow a consistent pattern in explaining why focusing on trust and not on satisfaction can change patients’ views on pharmacy services’ role. However, there is an implicit awareness that trust supports the principles of pharmaceutical care. For instance, in Portugal, pharmacies are highly trusted institutions, with significant agreement on the extension of pharmacy services [33]. Similarly, studies conducted in the UK [34] and Malta [35] found positive attitudes for the involvement of community pharmacies in activities promoting healthy living

Our research challenges conventional research on pharmacy services focused dominantly on patient satisfaction, aligning to the claims that this does not benefit the development of pharmaceutical care. Instead, building up trust is a path compatible with the creation of therapeutic relationships or alliances [36], strengthening patient-centered pharmacy practices. As such, we expand previous approaches in which trust was a simple predictor of patient satisfaction with pharmaceutical care [3,37,38] by exploring sets of determinants that allow targeted interventions to improve patient trust in the information received. Although satisfaction with pharmaceutical care and trust in the information received are highly correlated, we show that they, in fact, respond to different predictors that shape policy interventions in specific ways.

The Romanian context is relevant for this study for several reasons. Firstly, the population’s poor health status [39,40] commands a comprehensive understanding of all health structures and actors that can improve health outcomes. To the moment, most studies are focused on measuring satisfaction either with hospital services [41] or with public health services in general [42,43]. As for pharmacy services, current studies are more focused on the market dimension [44] and less on the determinants of satisfaction as such. This unexplored ground uncovers many potential opportunities. For instance, the high density of community pharmacies in Romania [45] signals a possible avenue to extend the reach of important primary health care services more cost-effectively. The context is also auspicious to such inquiries giving that an update on the legal framework of pharmaceutical services was made recently (law 243/6 November 2020), explicitly highlighting the principles of pharmaceutical care: a focus on providing the best response to patients’ needs, an approach that goes beyond selling medicines and includes a concern for their rational use, up to prevention campaigns and further personalized interventions.

Secondly, from an academic perspective, there is a research gap in identifying the determinants of satisfaction and trust at a local level. The authors in [46] offers some insights on what drives customer satisfaction with pharmaceutical providers through a transversal survey: age, sex, education level, ethnicity, residency, monthly income, marital status, number of family members, and presence of chronic disease appear as predictors of satisfaction. The study also emphasizes price sensitivity as the main reason to switch pharmacy providers, a result that flags a likely consumer-patient mindset [47,48]. This conclusion is further supported by the specific type of community pharmacy implemented in Central and Eastern Europe: the sort that provides all kinds of health care amenities [49] and is subject to direct-to-consumer pharmaceutical advertising [50]. Concerning trust, no Romanian studies examined the relationship between patients and community pharmacists, to the best of our knowledge.

## 2. Materials and Methods

### 2.1. Data

We collected data via an online questionnaire distributed between February 2020 and August 2020 and disseminated through Facebook, LinkedIn, WhatsApp, and by email. The study has been supported by the Romanian Alliance of Chronic Patients and has received the ethical committee approval No 03/08.01.2021 from the Scientific Research Ethics Committee of the University of Bucharest. The respondents were informed that their participation is voluntary and anonymous and that by completing the questionnaire, they provide consent to participation in this study. Our sample is the result of a combination between convenience sampling [51,52], meaning that the participants are self-selected based on their availability to answer the questionnaire, and snowball sampling [53,54], meaning that each participant passes the questionnaire to their social networks, thus acting as a seed for identifying new groups of respondents. Although this type of sampling is often criticized for not providing representative samples due to the non-representative typology of the Internet-recruited participants and to potential biases coming from self-selection effects [55,56], previous research shows that if the sample size is large enough, the final sample reaches similar structures regardless the initial distribution of seeds [54]. In addition, previous contributions advocate for caution in respect to the conclusions drawn from convenience sampling, rather for the rejection of the study based on such sampling method [57]. The participants did not receive any material compensation or prize for their participation in the research. 

### 2.2. Measurement

We measured the respondents’ degree of satisfaction with pharmaceutical services using a direct and an indirect approach. This type of measurement is documented in the literature [58] and was previously applied in Romania to assess patients’ satisfaction with public health services [43]. First, we measured overall satisfaction by asking the participants to rate their first-hand experience with community pharmacies. Then, we asked them to assess their satisfaction with aspects such as geographic proximity of the pharmacy, quality of interaction with pharmacists in terms of communication, waiting time, politeness and respect, the attention received, perceived reliability of information both in terms of instructions regarding the administration of the drugs, adverse effects, and possible interactions with food or other medication [24,59,60,61].

The items are presented in Table 1 and they serve as a formative basis for variables with a proven significant impact on the perceived customer value for community pharmacy services [62]. The perceived customer value further influences consumer satisfaction and consumer loyalty, with a more salient effect in pharmacy services by comparison with other service-intensive industries [24]. In addition, we also measured the level of trust in the advice received in pharmacies as a unidimensional construct [63].

As control variables (presented in Table 2), next to socio-demographic characteristics, we measured patients’ perception regarding the extent to which profit drives pharmacists’ recommendation for drugs [64]. This is a prevalent belief discussed in the literature as illustrating a conflict between ethics and business, conflict that lies amid issues like the legal recommendations for patient counseling, the community pharmacy’s economic objectives, and the morality of the pharmacist’s behavior [65,66]. In addition, we measured self-reported health status [67,68] in two different ways: comparative health status and health status.

### 2.3. Method

First, we used statistical tests to explore whether satisfaction with pharmacy services, and trust in information received in pharmacies differ by various socio-economic groups. To identify the appropriate tests, parametric or non-parametric, we conducted a preliminary analysis of the normality of these variables using the Shapiro–Wilk normality test and the D’Agostino skewness test (for a detailed presentation of the importance of these tests in normality exploration, see [69]). 

After concluding that satisfaction and trust are not normally distributed, we used the Wilcoxon rank sum test (the non-parametric equivalence of the *t*-test) to check whether there are differences by groups with two categories such as Gender and Education; the Kruskal–Wallis rank sum test (the non-parametric equivalence of ANOVA) to test for differences across more than two groups, in cases such as Civil status, or Income group. In addition, we used Spearman’s correlation (the non-parametric alternative to Pearson’s correlation) to test whether satisfaction and trust are related to numerical characteristics such as age, or whether they are related with each other. 

In stage 3, we conducted exploratory factor analysis to identify latent constructs among the independent variables presented in Table 1. Then we used robust regression analysis to fit two models that explain satisfaction with pharmaceutical services, and respectively trust in the information received from pharmacists, as predicted by the independent variables discussed above. The data analysis was conducted using the R statistical software, version 3.6.1 (R Foundation for Statistical Computing, Vienna, Austria).

## 3. Results

The minimum sample size for a power level of 95% and a significance level of 5% is 279 if calculated based on the inverse square root method, and 261 if calculated based on the gamma-exponential method [70]. We conducted the analysis pertaining for statistical power and sample size calculation in WarpPLS 7.0 [71]. Our data comprised 343 respondents, age between 18 and 79 years (mean = 47.42, median = 49, sd = 13.06). Only 23 participants had ages above 65, namely, 6.7% of the respondents. Out of the total, 84% of the respondents were women, 70% had higher education, 35% were members of a patients’ association, and 52.5% were chronic patients. Table 2 shows the complete socio-demographic characteristics of the sample. 

### 3.1. Patients’ Satisfaction with Services Received in Community Pharmacies

This subsection discusses the properties of the distributions of two variables central to our investigation: satisfaction with services and trust in the information received from pharmacists.

#### 3.1.1. Satisfaction with Services Provided in Pharmacies

A preliminary Shapiro–Wilk normality test shows that the distribution of patients’ overall satisfaction with pharmaceutical services is not normally distributed (W = 0.838, *p*-value < 0.001). A further D’Agostino skewness test shows a skewness value skew = −1.369 (*p*-value < 0.001), which is indicative of a significantly left-skewed distribution. Thus, there is a tendency towards high levels of satisfaction with pharmacy services.

Table 3 shows that socio-demographic factors such as age, level of education, gender, income group, or civil status are not associated with patients’ satisfaction with community pharmacies. Being a chronic patient is also a marginally relevant predictor. However, those who report going to the pharmacy more often than once a week tend to be more unsatisfied than the rest of the respondents. In addition, the level of trust in the information received in pharmacies positively relates to overall satisfaction, with a Spearman rho of 0.643. Self-reported health status is significantly related to general satisfaction, as follows: better health status is associated with higher levels of satisfaction in the absolute measurement, while testing for comparative health status shows once again a significant relationship. 

#### 3.1.2. Trust in the Information Received in Pharmacies

A preliminary Shapiro–Wilk normality test shows that the distribution of trust in the information received from pharmacists is not normally distributed (W = 0.897, *p*-value < 0.001). A further D’Agostino test shows a skewness value of −0.852 (*p*-value < 0.001), indicating a significantly left-skewed distribution and, therefore, high levels of trust reported by patients. However, compared with the distribution of overall satisfaction with pharmaceutical services, trust is less skewed and with lower kurtosis. This shows that the levels of trust are milder than satisfaction levels.

Table 4 shows that trust is correlated with age, older people reporting higher trust.

However, a visual inspection of the data shows that as age increases, the answers’ variability changes. Young people (18–22 years old) report higher trust levels, and so do older people (above 60).

Furthermore, we found that trust in information does not depend on gender, education, civil status, income group, or the frequency with which a respondent goes to the pharmacy. Chronic patients are marginally less confident in the advice received in pharmacies. Similar to the case of overall satisfaction, self-reported health status is significantly associated with trust: better health status is associated with higher levels of trust, while testing for comparative health status also shows statistical significance. 

#### 3.1.3. Determinants of Satisfaction and Trust

First, we checked each measurement’s internal consistency and found that the items related to pharmacy characteristics (PCH) show moderate reliability with a Cronbach’s Alpha of 0.69; 95%CI [0.64, 0.74]. Although the value is slightly below the recommended threshold of 0.7, it is still accepted in exploratory studies [72]. The Bartlett’s Test of Sphericity shows adequate value (X2 = 216.035, *p*-value < 0.001) and the Kaiser–Meyer–Olkin statistic is 0.67: a value above the recommended threshold, thus indicating moderate adequacy of the data for factor analysis. We extract a single factor that accounts for approximately 50% of the variability in data. Given the lack of normality in our data, we used the extraction method “pa”, and rotation “Promax”. The information is summarized in Table 5.

The quality of patient–pharmacist interaction was measured using nine items coded as PB in Table 2. The measurement has a very good internal consistency, which a Cronbach’s Alpha of 0.88 that can be improved if item eight (“In the interaction with me, the pharmacist was bored.”) is dropped. The Bartlett’s Test of Sphericity shows adequate value (X2 = 2166.137, *p*-value < 0.001) and the Kaiser–Meyer–Olkin statistic is 0.92, both values showing the appropriateness of factor analysis applied to this data. 

A preliminary parallel analysis recommends three factors, but after removing items 4 and 7 that load in more than one factor or have loadings higher than one, two factors remained that explain 63.6% of the variance in data. Table 6 shows that items 1, 5, and 6 load in the first factor standing for the pharmacist’s attitude, while the rest of the items stand for the pharmacist’s interest in their clients and the attention received by the patients.

The guidance received in pharmacies was measured using eight items (see Table 1), with an excellent internal consistency: a Cronbach’s Alpha of 0.94, that stays the same if items 7 and 8 are removed. The Bartlett’s Test of Sphericity shows adequate value, X2 = 2673.393, *p*-value < 0.001, and a very good value of the Kaiser–Meyer–Olkin statistic: 0.9. After removing items 7 and 8, the six remained items load in two different factors, as presented in Table 7.

The items that load in the first factor stand for information regarding possible side effects and potential drug and food interactions, a latent construct labeled as “precautionary information”, while the rest of the items load in the second factor, standing for guidance in drug administration. 

As the last step, we fit two regression models that explain satisfaction with pharmaceutical services on one side, and trust in pharmacists on another side, as a function of all predictors discussed until now. Table 3 and Table 4 show that the only socio-demographic variable related to both outcomes is health status, in absolute measurement, and in comparative measurement. Age is correlated with trust, while the frequency of visiting a pharmacy-related with satisfaction. First, we fit models with all variables, and then use backward regression to select the final models. Table 8 presents the results of two robust regression estimations. 

Table 9 summarizes the main results presented in Table 8, in a more intuitive way, showing only the direction (positive recorded as +) of the statistically significant relationships. In this presentation, the segregation of the determinants (except for Waiting time) becomes even more evident.

## 4. Discussion

Our study explores predictors of patient satisfaction with services and of trust in the information received in Romanian community pharmacies. Since Romania recently recognized officially, through explicit legislation, the transition towards a pharmaceutical care paradigm, an emphasis on trust and reliable information from official sources is essential to counteract a potential resistance to the changes involved by this transition, particularly a resistance to acknowledge pharmacists as important healthcare professionals [73,74]. To that purpose, we argue that to strengthen pharmacists’ widened role and effectively progress from providing simple indications to more complex recommendations [75], the uncertainty involved in the patient–pharmacist relationship, sometimes referred to as information asymmetry, needs to be addressed. We found that satisfaction with pharmaceutical services is indeed correlated with trust (Spearman’s rho = 0.643), a result that aligns with previous literature that discusses trust as a determinant of patient satisfaction in different health contexts [76,77], including pharmacy services [78,79]. Further, we expand the existing paradigm by showing that trust and satisfaction are predicted by different determinants, and thus they should be approached differently. In fact, we found that overall satisfaction with pharmaceutical services and the trust in the information received in pharmacies share only one predictor—waiting time, highly significant in both cases.

Satisfaction is significantly predicted by pharmacist’s attitude (β = 0.631, *p* < 0.001), waiting time (β = 0.180, *p* < 0.001), economic costs (β = 0.09, *p* = 0.009) and drug availability (β = 0.157, *p* < 0.001). These predictors are variables generally relevant for evaluation service/product quality and customer relationship management in a large range of business contexts, with implications for building customer loyalty and post-purchase behavior [80]. In this sense, of placing the pharmacy in the store satisfaction framework, our findings are consistent with previous research showing that pharmacist’s attitude determines the choice of a pharmacy among Romanians [44].

Trust in information received in pharmacy reveals a distinct story since, it is predicted by the attention received from pharmacist (β = 0.610, *p* < 0.001), and by the extent to which the pharmacist provides precautionary information regarding possible side effects and interactions of recommended medication with other drugs and food (β = 0.425, *p* < 0.001). By contrast to the business salient satisfaction’ determinants, this set of variables is meant to protect the potential vulnerability of patients, an emotional state usually involved in instances where trust and trustworthiness are required [79].

These antithetic results confirm our expectations that targeting patient satisfaction as a central objective may fail to address a core aspect that supports the expansion of the new pharmaceutical care. Without prioritizing trust, the focus only on patient satisfaction may bring just the illusion of further diminishing the information asymmetry in the patient–pharmacists [81] link, to the point of transforming completely this interaction in a market transaction. This will lead to a scenario in which community pharmacies do not move towards being better integrated in the public health system but in the business one. Fortunately, the literature indicates that building up trust is actually profitable also from a business point of view since it has been proved that trust in the information received in pharmacists drives satisfaction and further trust in pharmacies [78]. 

Another noteworthy result is the consistent difference observed in the perception of chronic versus non-chronic patients. Namely, chronic patients exhibit a lower level of trust in the information received in pharmacies (β = 0.328, *p* = 0.04). The difference may be potentially explained by the presumably closer relationship between chronic patients and family doctors or specialists (at least in terms of frequency of interaction), and a subsequent sense of patient loyalty combined with avoidance of other sources of information. However, this is not to say that trustworthy relationships cannot be developed in both instances, which would be ideal, but just to enunciate the necessity for explicit collaboration mechanisms between traditional health providers and pharmacists, in the effort of extending trust. A second explanation to be considered is the lack of dedicated information/health services/interventions for chronic conditions in pharmacies, by comparison to what is provided by family doctors or specialists. If this is the case, there is evidence to support that educating patients on pharmaceutical care’s prospects increases their interest in the matter [82], which is a good starting point. However, it is realistic to recognize that additional information does not completely change patient’s initial expectations on the role of pharmacists [83], respectively on their own role in issues like “responsible behavior”, “creating a patient-centered relationship”, and “interpersonal communication” [84]. Such behavioral and attitudinal changes are part of a long term process that needs consistent reinforcement of the patient–pharmacist relationship, consistent with other exploratory studies suggesting that “trust in pharmacists is more often than not earned than conferred” [85]. Concrete examples can be found in the extensive body of evidence showing the positive impact of community pharmacy interventions in areas like diabetes and hypertension management [86].

Not last, the focus on trust could also beneficial for non-chronic patients, in reinforcing rational medicine use and in offsetting the prevalent self-medication practices observed in Romania [87,88], especially with non-prescription (over-the-counter) medicines and traditional remedies [89]. The results obtained for chronic patients are far-reaching in light of the existing evidence showing that a more efficient path to patient adherence in medical care settings is achieved through trust rather than satisfaction [90]. Thus, increasing trust for this category of patients can generate improved medication access and better medication management [91]. This has augmented importance in the current context in which community pharmacies are considered “the most accessible group of health practitioners during this COVID-19 pandemic to address the substantial issues of inappropriate use of and promote rational use of medicines to people in the community as well as to special populations like patients with chronic conditions” [92].

The main limitation of our research comes from the convenience nature of our sample. Another limitation comes from the fact that the majority of our sample comprises people with higher education, which makes people with middle and elementary education under represented. The same applies to gender, where women are overrepresented. While we fully acknowledge the study’s exploratory nature, we also hope it will serve as a reference point for further research in this rather neglected healthcare system niche.

Secondary concerns are related to our overall satisfaction measurement for both satisfaction and trust, without considering different types of services and advices patients with different conditions may need. Although this is a common approach in the literature, and although we accounted for health status, and differentiated between chronic and non-chronic patients, both variables being good proxies for individuals requiring specific pharmacy services, further segmentation may be useful [93], on both individuals and services. 

Next to patient satisfaction with pharmacy services, the relationship pharmacist–patient is a decisive component in improving the system, deserving more empirical evidence. Future studies should look into the separate contribution of satisfaction and care to the intention and actual adoption of pharmaceutical care, and they should explore these variables as potential mediators of the relationship between socio-demographic characteristics, community pharmacy characteristics and patient–pharmacist relationship on one side, and the adoption of pharmaceutical care on another side. 

## 5. Conclusions

Our study compared the determinants of Romanian patients’ perception of community pharmacies, operationalized through satisfaction with services, and trust in information received. 

We expanded the existing literature on the dynamic and development of pharmacy services by taking a distinct data analysis perspective in showing that, although highly correlated, satisfaction with services and trust in information do not share the same predictors. Namely, market-related predictors drive satisfaction (pharmacists’ attitude, waiting time, cost of the drugs, and drug availability), while trust is explained by variables related to psychological comfort (pharmacists’ attention), the degree of information asymmetry experienced by patients (whether the patient received precautionary information), and whether the respondent is a chronic patient. 

Our results emphasize the importance of reducing information asymmetry in a patient–pharmacist relationship as a key direction for increasing patients’ acceptance and use of advanced services offered in pharmacies, as proposed by the pharmaceutical care paradigm. 

## Figures and Tables

**Table 1:** Items measuring patients’ satisfaction with pharmaceutical services and potential determinants.

Dimension	Item Abbreviation	Item
Outcome variables		
General satisfaction	GS	On a scale of 1 to 9, where 1 means Very dissatisfied and 9 means Fully satisfied, how satisfied are you in general with the services offered to you in pharmacies?
Patients’ trust in the advice received in pharmacies	TRUST	On a scale of 1 to 9, where 1 means Very little and 9 means A lot, how much confidence do you have in the advice you receive in pharmacies?
Independent variables		
On a scale from 1 to 7, where 1 means Total disagreement, and 7 means Total agreement, what is your level of agreement with the following statements?		
Dimension	Item abbreviation	Item
Pharmacy characteristics	PCH1	The position of the pharmacy is convenient for me.
	PCH2	The waiting area in the pharmacy is comfortable and convenient.
	PCH3	The pharmacy is very clean.
Costs and drugs availability	CDA1	The cost of the drugs that I need is reasonable.
	CDA2	The medication I need is available according to my needs.
Waiting time	TIME	The pharmacy staff is sufficient to serve customers in a reasonable time.
Pharmacist’s behaviour	PB1	The pharmacist was polite.
	PB2	The pharmacist was interested in my needs.
	PB3	The pharmacist treats all customers the same.
	PB4	The pharmacist treats customers with respect.
	PB5	The pharmacist was available during my visit.
	PB6	The tone that the pharmacist used was polite.
	PB7	The time it took the pharmacist to process a prescription was reasonable.
	PB8	In the interaction with me, the pharmacist was bored.
	PB9	The amount of time spent by the pharmacist offering me medication advice was enough.
Guidance received by patient	GUID1	The pharmacist constantly stressed the importance of taking the medication as recommended.
	GUID2	The pharmacist gave me information on how to store/keep the drugs correctly.
	GUID3	The pharmacist gave me adequate information about the precautions to take when taking the drugs.
	GUID4	The pharmacist gave me adequate information about the side effects that the drugs can cause.
	GUID5	The pharmacist gave me adequate information about possible interactions between my medication and other drugs.
	GUID6	The pharmacist gave me adequate information about the possible interactions between my medication and some foods.
	GUID7	The pharmacist gave me clearly written instructions on how to take the drugs.
	GUID8	The pharmacist explained to me the details of taking the drugs in clear language.
Ethic dimension	ETHIC	Being a pharmacist involves both being a professional and a seller to what extent do you believe that the need for profit drives a pharmacist’ recommendations for drugs?

**Table 2:** Descriptive statistics of the sample.

Categorical Descriptors		Frequency			
Gender Female Male		83.7% 16.3%			
Education					
At most secondary education		30%			
Higher education		70%			
Civil Status					
Married		65.3%			
Single		28.9%			
Widow		5.8%			
Economic status					
Much below the average salary		16.9%			
Slightly below the average salary		14.0%			
Around the average salary		24.5%			
Slightly above the average salary		30.6%			
Much above the average salary		14.0%			
Patient associations membership					
Yes		35%			
No		65%			
The drugs purchased were:					
Subsidized		5.8%			
Out of pocket		58.9%			
Both subsidized and out of pocket		35.3%			
Self-reported chronic patient					
Yes		52.5%			
No		47.5%			
Numerical descriptors					
Variable	Min	Mean	Median	Max	SD
Age	18	47.42	49	79	13.06
Self-reported health status (measured 1–10)	1	7.306	8	10	1.9

**Table 3:** Tests that explore contingencies with overall satisfaction.

Satisfaction by:	Test	Test Results	Conclusion
Age	Spearman’s rank correlation	rho = 0.033 *p* = 0.547	Satisfaction does not depend on age
Gender	Wilcoxon rank sum test	W = 7516.5, *p* = 0.429	Satisfaction does not depend on gender
Education	Wilcoxon rank sum test	W = 13,242, *p* = 0.278	Satisfaction does not depend on education
Civil status	Kruskal–Wallis rank sum test	chi-sq = 1.3215, *p* = 0.516	Satisfaction does not depend on civil status
Frequency	Kruskal–Wallis rank sum test	chi-sq = 12.354 *, *p* = 0.015	Those who go to pharmacy more often tend to be less satisfied
Income group	Kruskal–Wallis rank sum test	chi-sq = 0.722 *p* = 0.949	Satisfaction does not depend on income
Chronic patients	Wilcoxon rank sum test	W = 13,168, *p* = 0.090	Chronic patients are marginally less satisfied than non-chronic patients
Trust in information received	Spearman’s rank correlation	Rho = 0.643 *** p < 0.001	Satisfaction with services and trust in information received from pharmacist are strongly correlated
Self-reported health status	Spearman’s rank correlation	rho = 0.240 *** p < 0.001	People with better health status (direct measurement) are more satisfied than people with poorer health status
Comparative health status	Kruskal–Wallis rank sum test	chi-sq = 8.385 * p = 0.015	People with better health status (comparative measurement) are more satisfied than those with poorer health status.

* *p* < 0.05, ** *p* < 0.01, *** *p* < 0.001.

**Table 4:** Tests that explore contingencies with trust in the information received in pharmacies.

Satisfaction by:	Test	Test Results	Conclusions
Age	Spearman’s rank correlation	rho = 0.124 * p-value = 0.021	As age increases, so does the trust in information received from pharmacist
Gender	Wilcoxon rank sum test	W = 7326.5, *p*-value = 0.288	Trust in information received does not depend on gender.
Education	Wilcoxon rank sum test	W = 13,252, *p*-value = 0.281	Trust in information received does not depend on education
Civil status	Kruskal–Wallis rank sum test	chi-sq = 2.165, *p*-value = 0.339	Trust in information received does not depend on civil status
Frequency	Kruskal–Wallis rank sum test	chi-sq = 5.063, *p*-value = 0.281	Trust in information received does not depend on the frequency of visiting the pharmacy
Income group	Kruskal–Wallis rank sum test	chi-sq = 3.954 *p*-value = 0.412	Trust in information received does not depend on income
Chronic patients	Wilcoxon rank sum test	W = 12,972, *p*-value = 0.06	Chronic patients are marginally less inclined to trust the advice received in pharmacies
Self-reported health status	Spearman’s rank correlation	rho = 0.236 *** p-value < 0.001	Better health status (direct measurement) is associated with higher levels of trust
Comparative health status	Kruskal–Wallis rank sum test	chi-sq = 10.034 ** p-value = 0.007	Better health status (comparative measurement) is associated with higher levels of trust

* *p* < 0.05, ** *p* < 0.01, *** *p* < 0.001.

**Table 5:** Factor loadings for the latent construct that accounts for pharmacy characteristics.

Item	Manifest Variable	Pharmacy Characteristics Factor Loadings
PCH1	The position of the pharmacy is convenient for me.	0.602
PCH2	The waiting area in the pharmacy is comfortable and convenient.	0.795
PCH3	The pharmacy is very clean.	0.669
Amount of variance explained: 48.1%		

**Table 6:** Factor loadings for the dimension related to pharmacist’s behavior.

Item	Manifest Variables	Attitude	Attention Received
PB1	The pharmacist was polite.	0.839	
PB2	The pharmacist was interested in my needs.		0.479
PB3	The pharmacist treats all customers the same.		0.949
PB5	The pharmacist was available for me during my visit.	0.472	
PB6	The tone used by the pharmacist was kind.	0.992	
PB9	The amount of time spent by the pharmacist offering me medication advice was sufficient.		0.661
Amount of variance explained: 63.6%			

**Table 7:** Factor loadings for two dimensions of the guidance provided by pharmacists.

Item	Manifest Variables	Precautionary Information	Drug Administration
GUID1	The pharmacist constantly stressed the importance of taking the medication as recommended.		0.705
GUID2	The pharmacist gave me information on how to store/keep the drugs correctly.		0.558
GUID3	The pharmacist gave me adequate information about the precautions to take when taking the drugs.		0.716
GUID4	The pharmacist gave me adequate information about the side effects that the drugs can cause.	0.776	
GUID5	The pharmacist gave me adequate information about possible interactions between my medication and other drugs.	0.991	
GUID6	The pharmacist gave me adequate information about the possible interactions between my medication and some foods.	0.838	
Amount of variance explained: 63.8%			

**Table 8:** Robust regression estimation of satisfaction and trust.

Model	Satisfaction	Trust
Intercept	5.317 *** (*p* < 0.001)	5.169 *** (*p* < 0.001)
Attitude	0.631 *** (*p* < 0.001)	-
Attention	-	0.610 *** (*p* < 0.001)
Precautions	-	0.425 *** (*p* < 0.001)
Position	-	0.092 (*p* = 0.437)
Age	-	0.009 (*p* = 0.127)
Cost	0.09 ** (*p* = 0.009)	-
Drug availability	0.157 *** (*p* < 0.001)	-
Chronic patient	-	Reference
Yes		0.328 *
No		(0.04)
Ethic	-	−0.064 (*p* = 0.2)
Waiting time	0.180 *** (*p* < 0.001)	0.287 *** (*p* < 0.001)

* *p* < 0.05, ** *p* < 0.01, *** *p* < 0.001.

**Table 9:** A side-by-side presentation of the results.

Model	Satisfaction	Trust
Attitude	+	
Attention		+
Precautions		+
Position		No significant relationship
Age		No significant relationship
Cost	+	
Drug availability	+	
Chronic patient Yes No		+
Ethic		No significant relationship
Waiting time	+	+

## Data Availability

Data is available on request.
